# The value of contrast-enhanced ultrasonography in the diagnosis of primary testicular non-neoplastic and neoplastic lesions in adults

**DOI:** 10.1186/s12894-022-01163-9

**Published:** 2022-12-22

**Authors:** Nianyu Xue, Shengmin Zhang, Guoyao Wang

**Affiliations:** Department of Ultrasonography, Ningbo First Hospital, 59 Liu Ting Street, Ningbo, 315010 Zhejiang China

**Keywords:** Conventional ultrasonography, Contrast-enhanced ultrasonography, Non-neoplastic lesions, Testicular tumor

## Abstract

**Background:**

Different pathological types of testicular tumors are treated differently. Malignant germ cell tumors require radical orchiectomy, while benign tumors may only need mass enucleation. Contrast-enhanced ultrasonography (CEUS) is more sensitive than conventional ultrasonography in displaying tumor microvessels, which helps distinguish between benign and malignant tumors.

**Methods:**

This was a retrospective analysis of 35 patients with pathological-confirmed primary testicular non-neoplastic and neoplastic lesions in our hospital from February 2017 to February 2022. Conventional ultrasonography and CEUS imaging findings of included lesions were retrospectively analyzed and their diagnostic values were compared with the pathological results.

**Results:**

There were 13 cases of benign testicular lesions (including 1 case of spontaneous hematoma, 2 cases of segmental infarctions, 5 cases of epidermoid cysts, 2 cases of Sertoli cell tumors, and 3 cases of Leydig cell tumors) and 23 cases of malignant testicular lesions (including 10 cases of seminomas, 1 case of embryonal carcinoma, 2 cases of mixed germ cell tumors, 2 cases of spermatocytic tumors, and 8 cases of lymphomas). The sensitivity, specificity, positive predictive value, negative predictive value and accuracy rates of conventional ultrasound in diagnosing benign testicular tumors by "onion skin-like" echo (epidermoid cysts) and peripheral annular blood flow were 30.8%, 100.0%, 100.0%, 71.9% and 75.0%, respectively. All testicular non-neoplastic lesions and epidermoid cysts showed no enhancement by CEUS. All Sertoli-Leydig cell tumors (SLCTs)’ CEUS imaging showed uniform high enhancement (no necrosis area), fast forward, and slow backward. 80.0% (12/15) malignant germ cell tumors showed heterogeneous enhancement and fast forward and fast backward in CEUS. All lymphomas showed fast forward and fast backward, and 87.5% (7/8) of them showed uniform high levels of enhancement in CEUS. According to CEUS without enhancement (non-neoplastic lesions and epidermoid cysts) and uniform high enhancement with fast forward and slow backward (SLCT), the sensitivity, specificity, positive predictive value, negative predictive value and accuracy rates for diagnosing benign testicular tumors were all 100.0%. Compared with conventional ultrasound, the difference was statistically significant (*p* = 0.004).

**Conclusions:**

CEUS could accurately distinguish between benign and malignant testicular tumors, as well as differentiate specific pathological types (testicular focal infarction, epidermoid cysts, spermatocytic tumors, SLTC and lymphoma). Accurate preoperative diagnosis is critical for guiding the selection of appropriate treatment plans for different pathological types of testicular tumors.

## Introduction

Testicular tumors are the most common malignant tumors in adolescents and are classified into germ cell tumors (including seminoma, embryonal carcinoma, yolk sac tumors, trophoblastic tumors, teratoma, mixed germ cell tumors, spermatocytic tumors and epidermoid cysts), sex cord-stromal tumors (Leydig cell tumors and Sertoli cell tumors), and some other rare types of tumors. Most testicular tumors are germ cell tumors [1, 2]. In the past, most testicular tumors were considered malignant (with seminomas being the most common) [3, 4]. Needle biopsy is generally not recommended for testicular tumors as it can lead to local recurrence. Under the current treatment plan for testicular tumors, most testicular tumors (including benign ones) are subjected to radical orchiectomy, which will inevitably lead to infertility, psychological problems and endocrine disorders [5]. Testicular non-neoplastic lesions (hematoma, necrosis), benign testicular tumors (Leydig cell tumors), and lymphoma do not require radical orchiectomy. With the ability to detect small benign testicular masses, ultrasound has become the imaging modality of choice for scrotal disease [2, 6, 7]. However, relative to the high sensitivity of ultrasound to detect testicular masses, the diagnostic specificity is low. If radical orchiectomy is performed on all patients with testicular tumors, the testicles of some patients with benign testicular tumors are prone to be mistakenly incised. In this study, we retrospectively analyzed conventional ultrasonography and contrast-enhanced ultrasonography (CEUS) of primary testicular non-neoplastic and neoplastic lesions in adults, in order to provide an accurate preoperative diagnosis to guide clinical treatment and avoid unnecessary orchiectomy.

## Methods

### Subjects

This was a retrospective analysis of 35 patients with pathological-confirmed primary testicular non-neoplastic and neoplastic in our hospital from February 2017 to February 2022. The inclusion criteria were as follows: (1) testicular tumor and segmental infarction confirmed by histopathology and hematoma confirmed by cytopathology, (2) with complete routine ultrasound and CEUS data, (3) ages ≥ 18 years. The exclusion criteria were as follows: (1) incomplete data (i.e., lack of histological and pathological results and/or incomplete ultrasound images); (2) patients received non-steroidal anti-inflammatory drugs, radiotherapy, chemotherapy or other forms of immunotherapy, and (3) patients’ age < 18 years.

According to the pathological results, the patients were divided into two groups: (1) benign testicular lesions group, including non-neoplastic lesions (hematoma and segmental infarction), epidermoid cysts, Sertoli-Leydig cell tumor (SLCT); and (2) malignant testicular tumors group, including malignant germ cell tumor and lymphoma.

### Equipment and agents

The contrast agent used in CEUS was SonoVue (Bracco SpA, Milan, Italy). The agent consisted of microbubbles containing sulfur hexafluoride (SF 6) encapsulated in phospholipids. The microbubbles had a mean diameter of 2.5 μm and pH values of 4.5–7.5. The SonoVue powder was thoroughly dissolved in 5 mL normal saline; then, 2.4 mL of the solution was injected into the bolus through the cubital vein.

The ultrasound devices used included Aplio500 (TOSHIBA CORPORATION, Tokyo, Japan), LOGIQ E9 GE (General Electric Company, Boston, MA, USA), EPIQ7 (Philips Electronic N.V, Amsterdam, The Netherlands), EUB-8500 (HITACHI, Tokyo, Japan), and Aixplorer (SuperSonic Imagine, Aix-en-Provence, France). The CEUS function was available on all these devices. A linear array probe was selected with a frequency of 5.0–12.0 MHz for small masses, while an abdominal convex array probe was used with a frequency of 3.0–5.0 MHz for large masses, as the linear array probe may not be fully displayed.

### Methodology

Conventional ultrasound and CEUS images of the primary testicular non-neoplastic and neoplastic lesions were retrospectively analyzed. The parameters of lesion in the conventional ultrasound images included location, number, size, shape, echo, boundary, and color doppler flow imaging (CDFI). The "Onion skin-like" echo was used as a diagnostic criterion for epidermoid cysts. The peripheral annular blood flow was used as the diagnostic criterion for SLCT and the perforating vessel was used as the diagnostic criteria for lymphoma. If the testicular tumor is hypoechoic with abundant blood flow and irregular blood flow distribution, it is diagnosed as malignant germ cell tumor. The findings were interpreted by two physicians with 10 years of experience in scrotal ultrasound and each preliminary diagnosis was made after the physicians reached an agreement. The parameters of lesion in the CEUS included enhancement time, enhancement level (high, equal, low, or none) and contrast-agent distribution (uniform or non-uniform). The non-enhancement in the CEUS was used as a diagnostic criterion for testicular non-neoplastic lesions and epidermoid cysts. CEUS showing uniform high enhancement (no necrosis area) with fast forward and slow backward was used as the diagnostic criterion for SLCT. CEUS showing uniform hyperenhancement with fast forward and fast backward was used as the diagnostic criterion for lymphoma. CEUS showing heterogeneous enhancement was used as the diagnostic criterion for malignant germ cell tumors of the testis. The findings were interpreted by two physicians with five years of experience in scrotal ultrasound and each preliminary diagnosis was made after the physicians reached an agreement. Single blind method was used for diagnosis.All four physicians were blinded to the final diagnoses and other information at the time of image interpretation and preliminary diagnoses.

### Statistical analysis

Statistical analysis was performed using the SPSS 20.0 software package (Chicago, IL, USA). Count data were analyzed using the paired χ^2^ test and the diagnostic accuracy of the examinations was assessed by contingency tables. Measurement data were expressed as the mean ± standard deviation (SD). One-way analysis of variance (ANOVA) was used for comparisons among multiple groups and the LSD-t test was used for comparison between groups. A *p*-value < 0.05 was considered statically significant.

## Results

### Pathological and clinical findings

1. Pathological results: 35 patients with 36 masses were included. Except for one case of spontaneous hematoma confirmed by cytology, the other 35 cases of primary testicular lesions were confirmed by surgery or biopsy histopathology. There were 13 cases of benign testicular lesions (including 1 case of spontaneous hematoma, 2 cases of segmental infarctions, 5 cases of epidermoid cysts, 2 cases of Sertoli cell tumors and 3 cases of Leydig cell tumors), and 23 cases of testicular malignant lesions (including 10 cases of seminomas, 1 case of embryonal carcinoma, 2 cases of mixed germ cell tumors, 2 cases of spermatocytic tumors and 8 cases of lymphomas). Except for one case of bilateral testicular lymphoma, the others were single. 20 cases were in the right testis and 15 cases were in the left testis.
2. Clinical manifestations: Two cases of epidermoid cysts and two cases of Leydig cell tumors were asymptomatic. One case of spontaneous hematoma and two cases of segmental infarction showed dull pain in the testis. The remaining cases were all due to scrotal enlargement (one lymphoma and one epidermoid cyst with scrotal pain, the rest were painless enlargement).
3. Laboratory examinations: AFP and HCG were elevated in two mixed germ cell tumors; HCG was elevated in one seminoma; AFP was elevated in one embryonal carcinoma; and follicle-stimulating hormone and luteinizing hormone were elevated in one bilateral lymphoma. No obvious related abnormalities were detected.

### Conventional ultrasound and CEUS findings

The characteristics of conventional ultrasound and CEUS in benign and malignant testicular lesions are shown in Tables 1 and 2. There were statistically significant differences in lesion size, blood flow and CEUS enhancement between benign and malignant testicular lesions, while there was no statistical difference in the rest characteristics (*p* < 0.05) (Tables 1, 2).

**Fig. 1:**
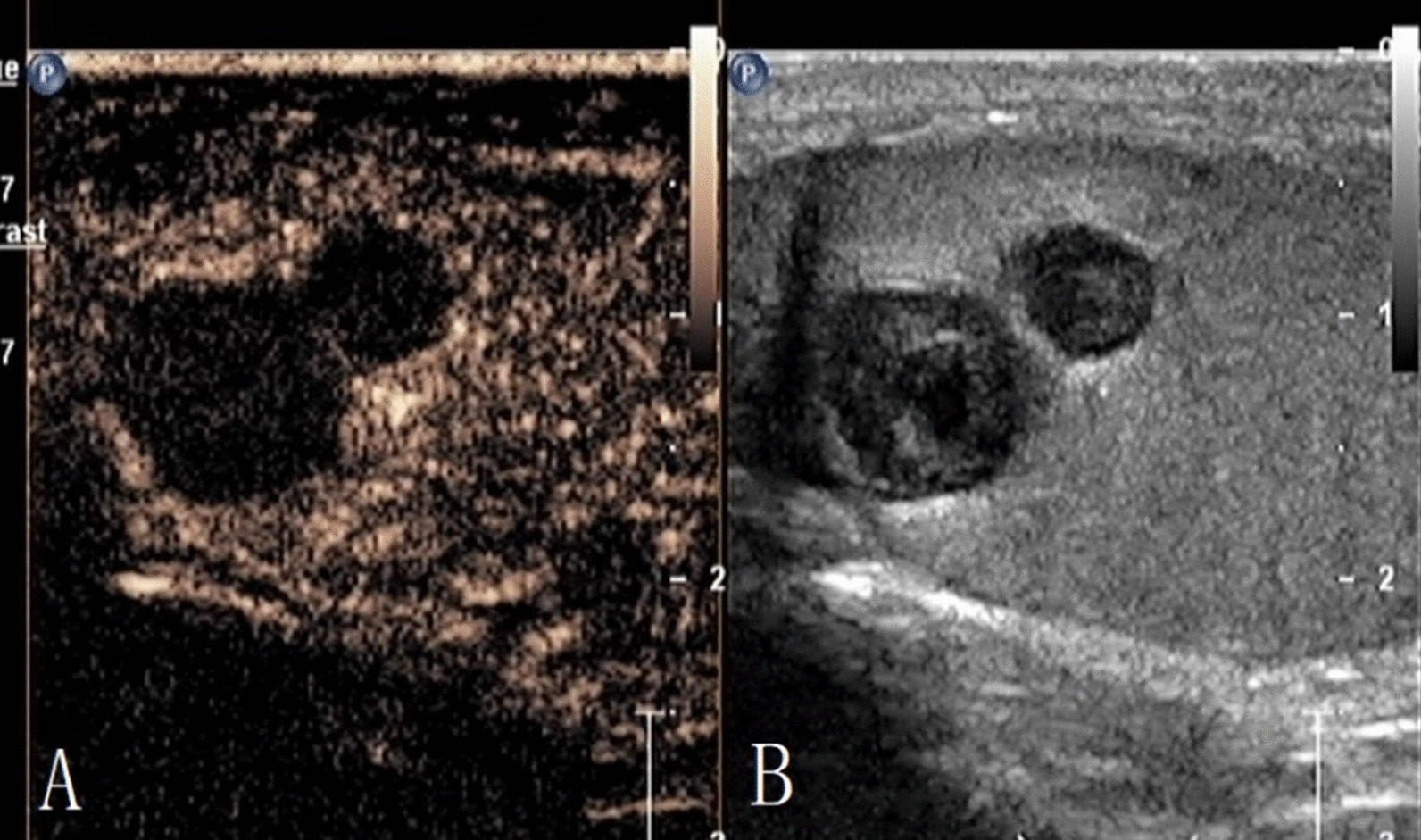
Testicular spontaneous hematoma. **A** Contrast-enhanced ultrasonography showed no enhancement in the mass and a diagnosis of spontaneous hematoma was made. **B** Conventional ultrasound showed that the right testis had clear boundary and irregular hypoecho. Because there was no history of trauma, this was considered to be malignant tumor

**Fig. 2:**
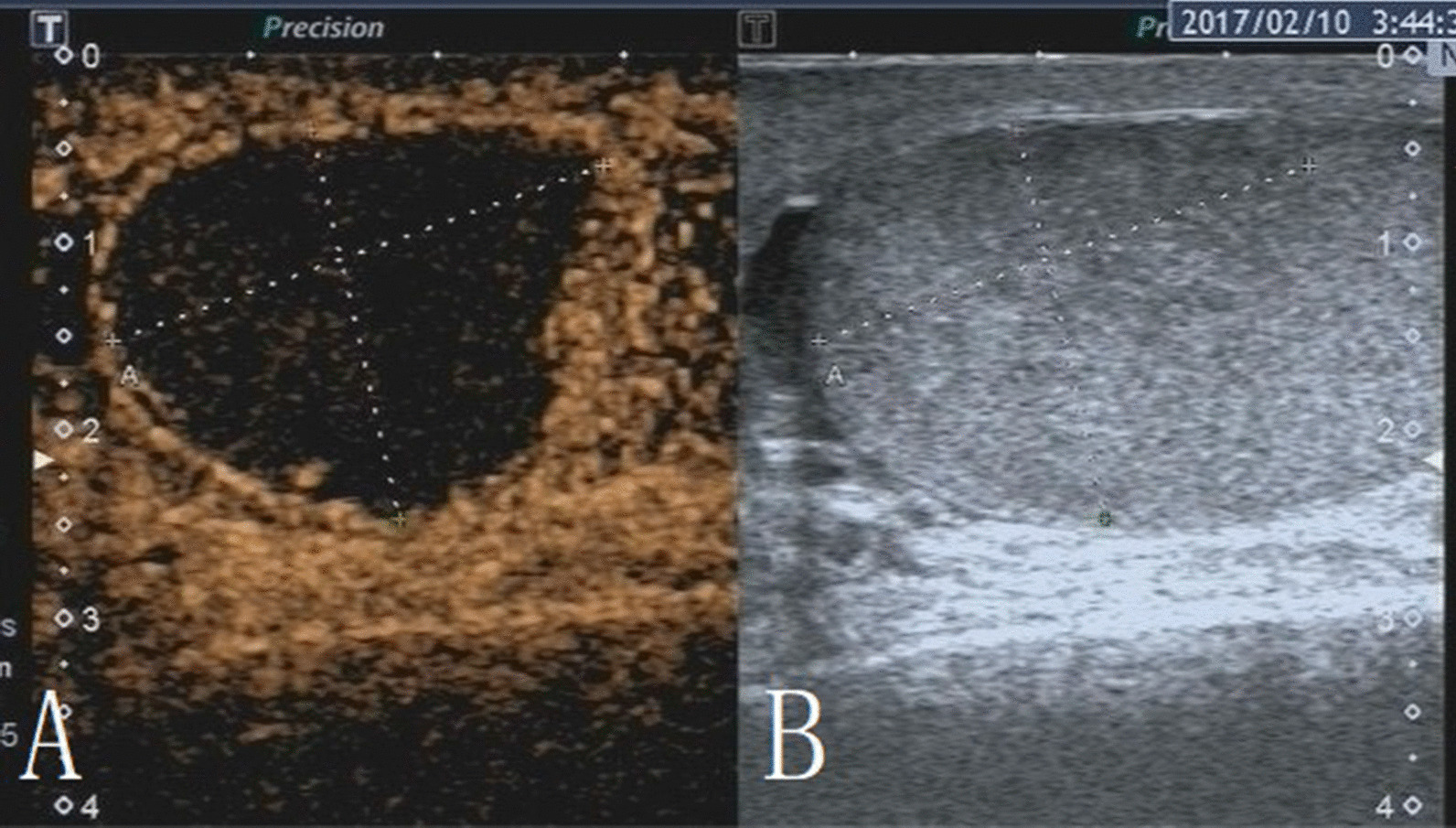
Testicular segmental infarction. **A** Contrast-enhanced ultrasonography showed no enhancement; the diagnosis was segmental infarction. **B** Conventional ultrasonography showed ill-defined iso-echo signals in the middle and upper part of the left testis. It was impossible to determine whether this was a tumor or not

**Fig. 3:**
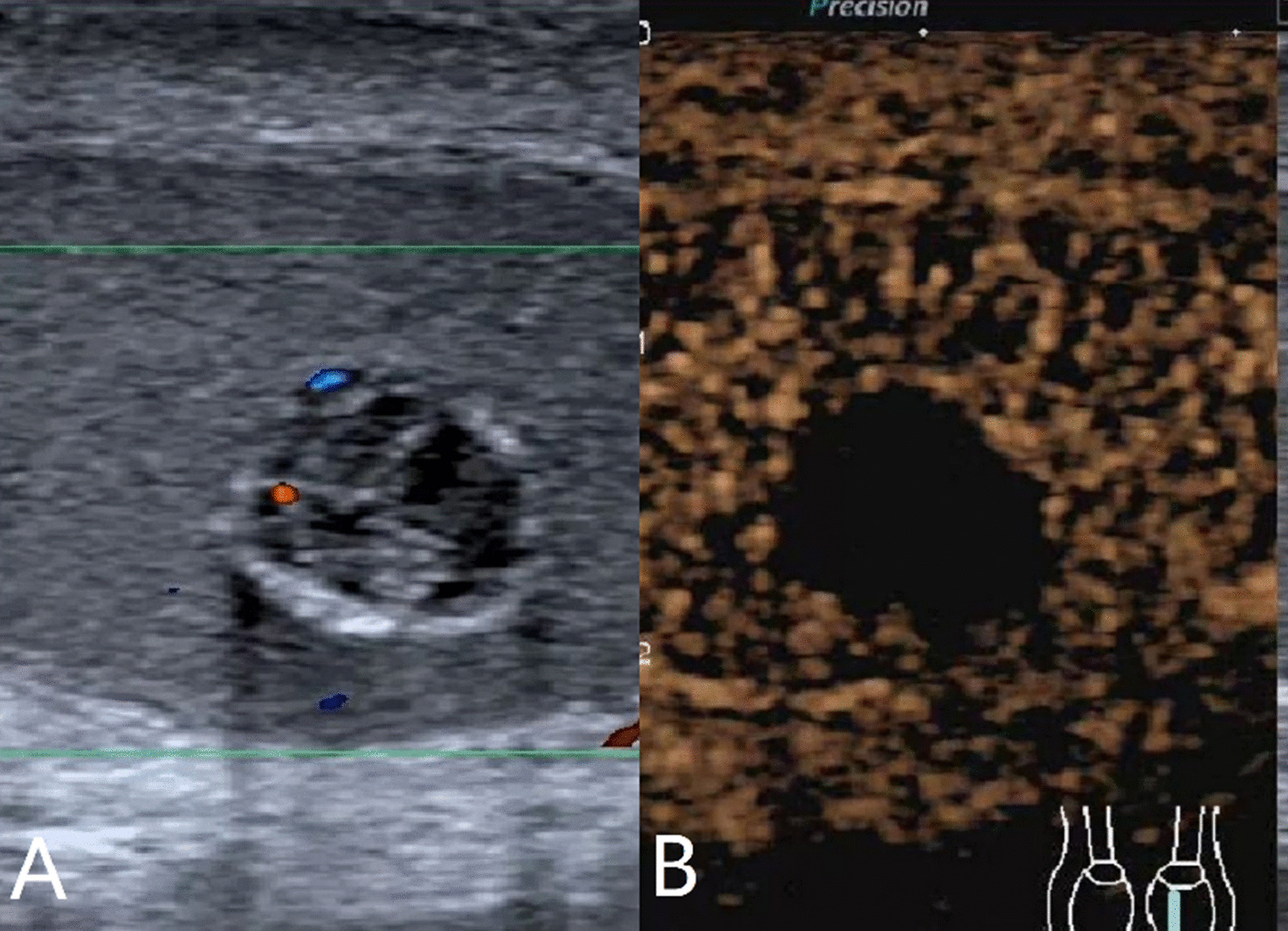
Testicular epidermoid cyst. **A** Conventional ultrasound showed an uneven echo in the left testis, calcification in the periphery and blood flow inside. This was considered to be a malignant tumor. **B** CEUS showed no enhancement; the diagnosis was epidermoid cyst

**Fig. 4:**
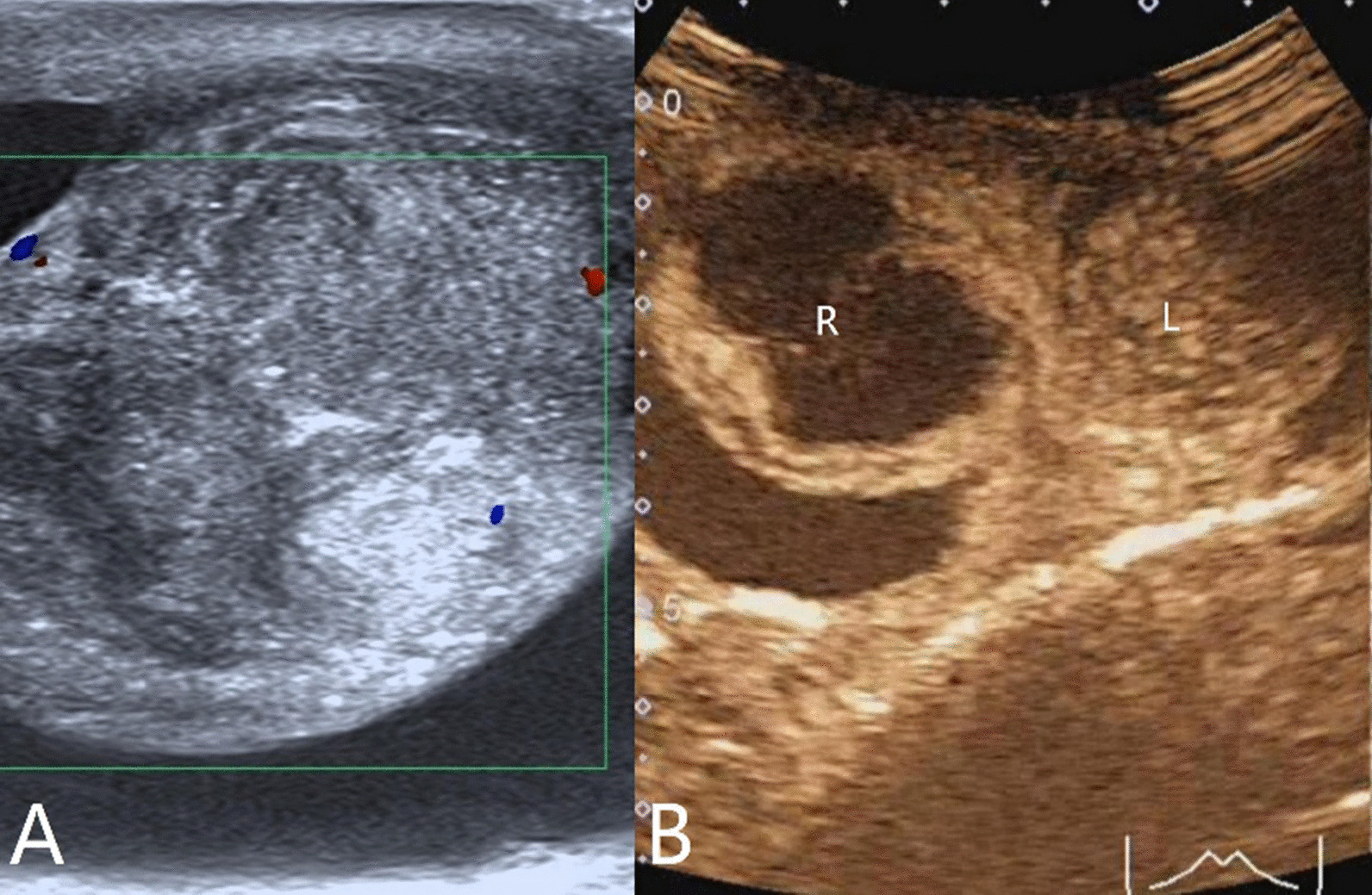
Testicular embryonal carcinoma. **A** Conventional ultrasonography showed that the right testis had an unclear boundary, irregularly shaped and uneven hypoechoic signals, and a little blood flow around it. **B** A large area of necrosis was evident in the center of the CEUS with high enhancement in the periphery. R: Right testis; L: left testis

**Fig. 5:**
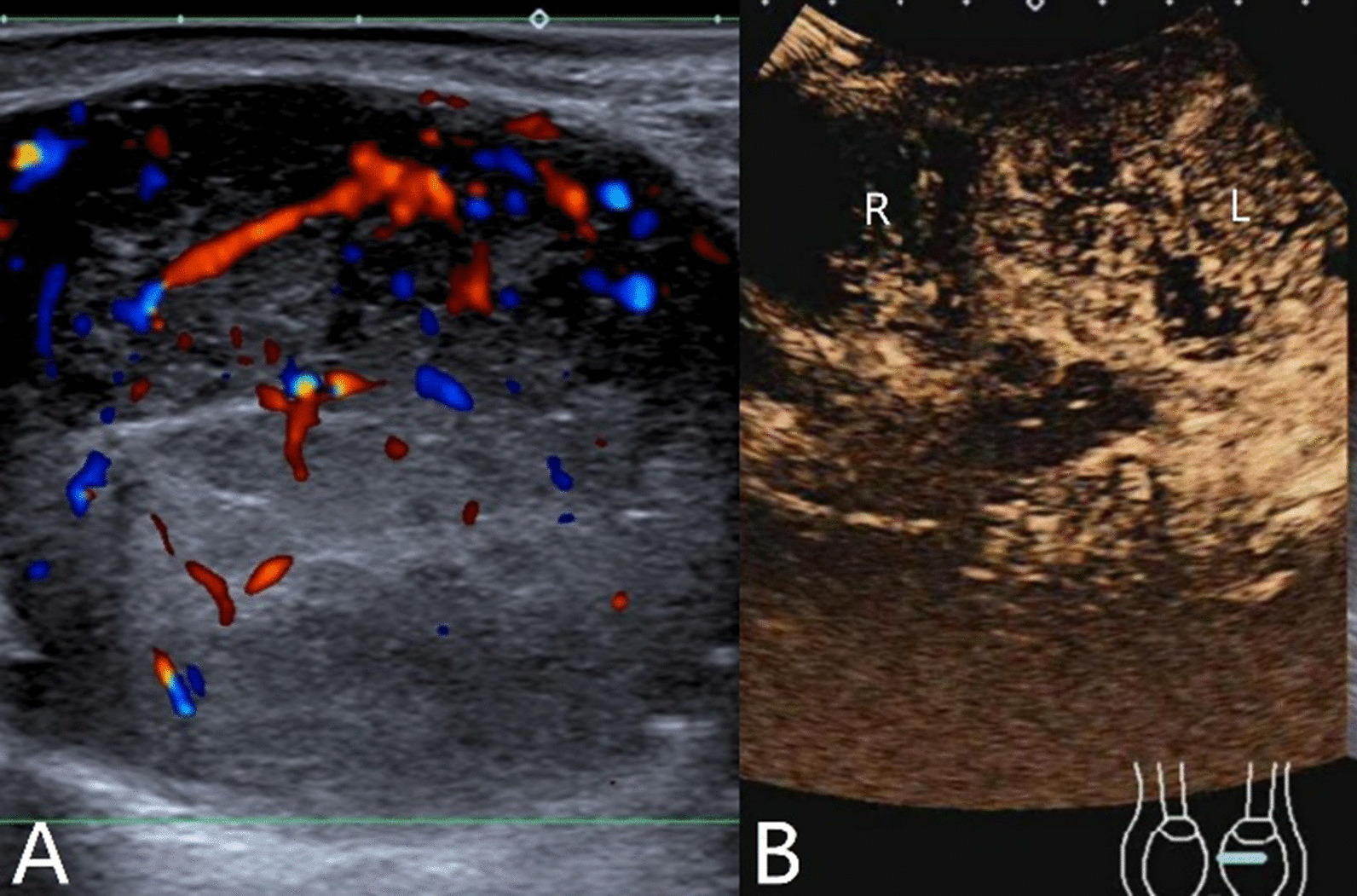
Testicular seminoma. **A** Conventional ultrasonography showed a round-like hypoecho with a clear border in the left testis and abundant blood flow. **B** Contrast-enhanced ultrasonography showed non-homogeneous hyperenhancement with necrotic areas

**Fig. 6:**
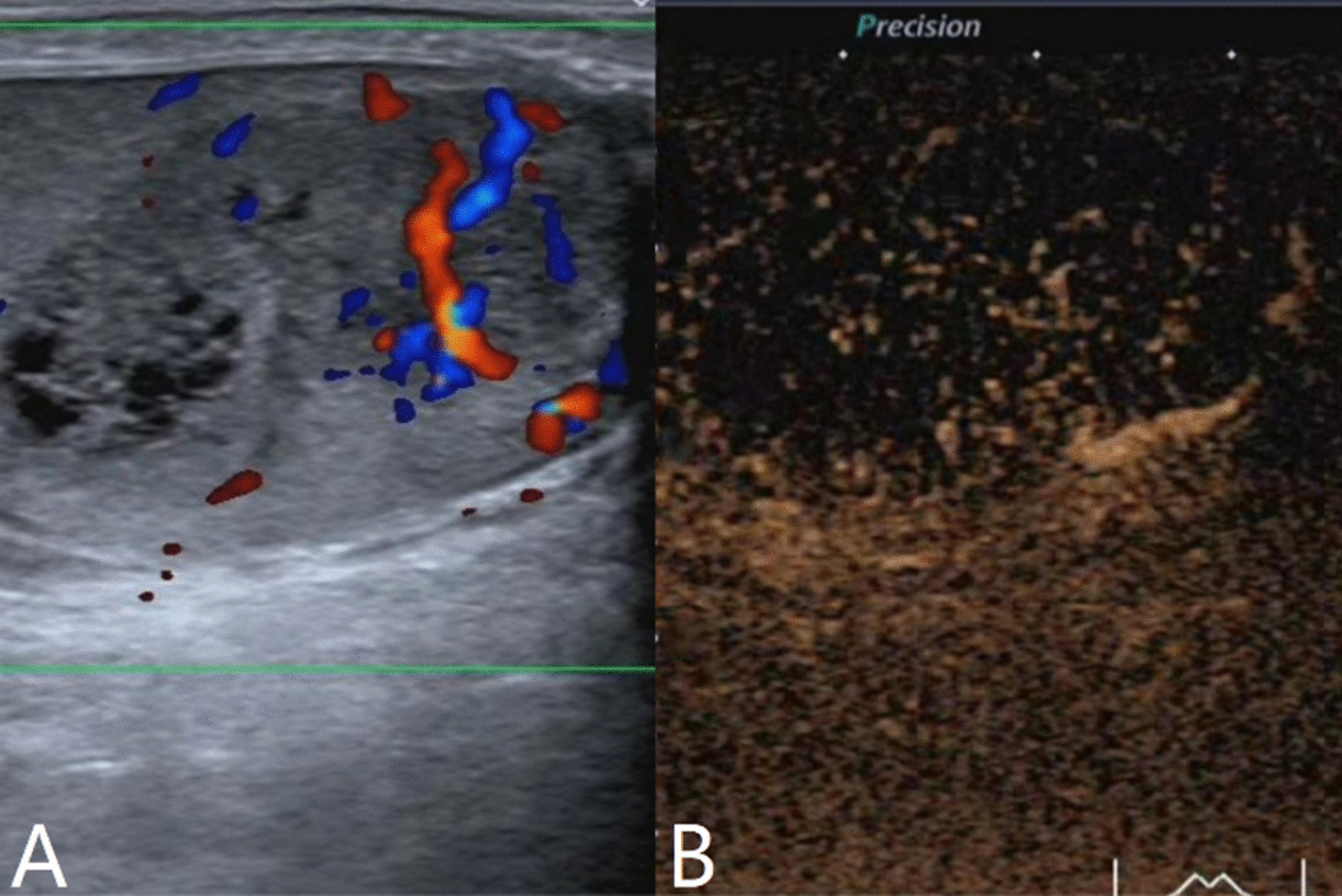
Testicular seminoma. **A** Conventional ultrasonography showed cystic and solid mixed echoes with clear borders in the right testis; the solid part was rich in blood flow. **B** Contrast-enhanced ultrasonography showed sparse and low enhancement

**Fig. 7:**
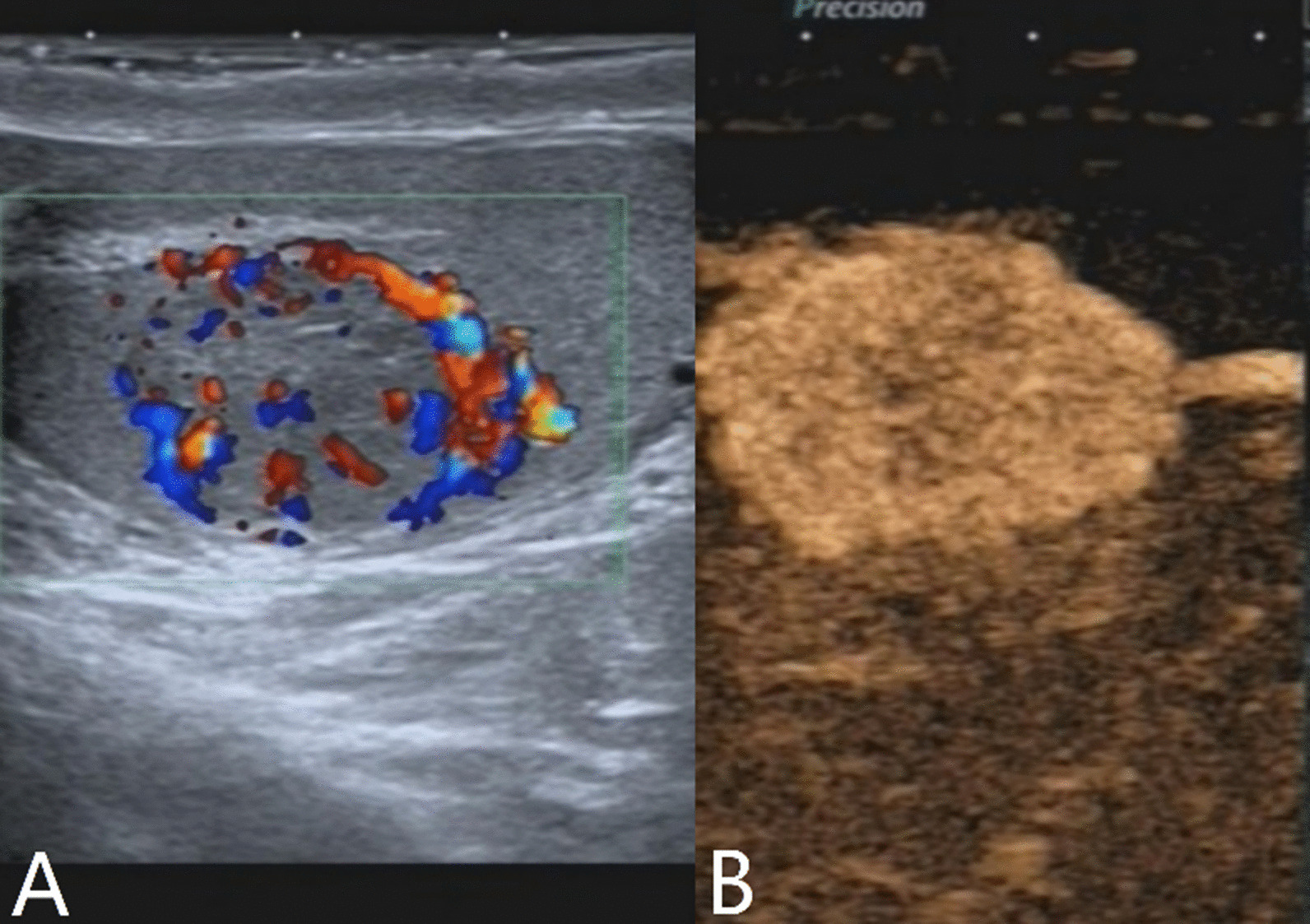
Testicular Leydig cell tumor. **A** Conventional ultrasonography showed that the right testis had a clear isoechoic boundary with abundant blood flow and peripheral annular blood flow. **B** Contrast-enhanced ultrasonography showed uniform high enhancement

**Fig. 8:**
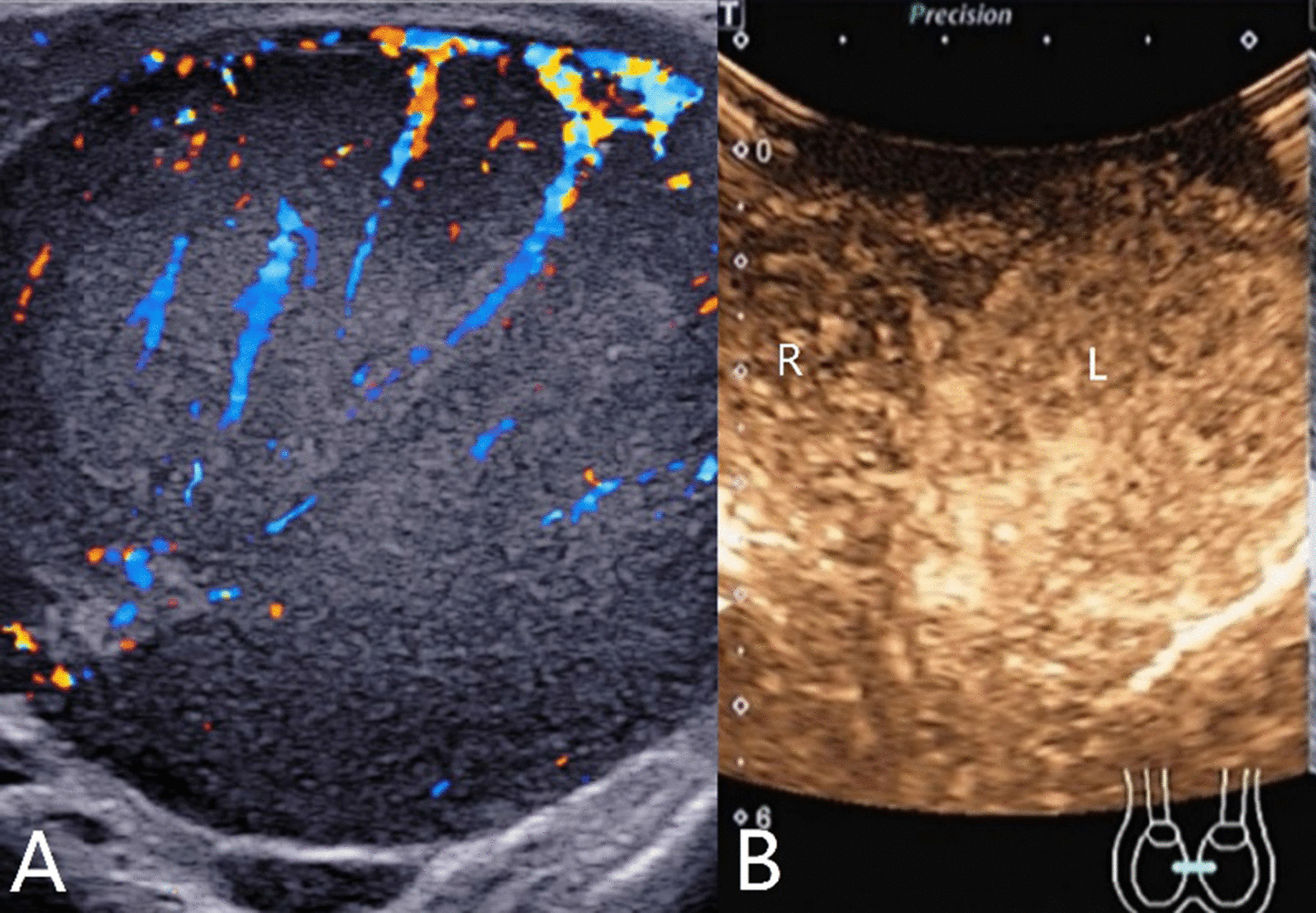
Testicular lymphoma. **A** Conventional ultrasonography showed that the left testis had a clear and hypoechoic border with abundant blood flow inside. **B** Contrast-enhanced ultrasonography shows uniform high enhancement. R: Right testis; L: left testis

1. Testicular non-neoplastic lesions: Since there was no history of trauma, one case of spontaneous hematoma was misdiagnosed as malignant by conventional ultrasound (Fig. 1). In two cases of segmental infarctions, conventional ultrasound could not determine whether it was a tumor or not (Fig. 2). The CEUS showed no enhancement in all three cases (Figs. 1, 2) leading to the possibility of segmental infarction or spontaneous hematoma.
2. Epidermoid cysts group: CDFI showed that except one case had no blood flow signal (diagnosed as an epidermoid cyst due to an "onion skin-like" echo), the other four cases had blood flow signals (possibly a twinkle artifact) and were diagnosed as malignant tumors by conventional ultrasonography (Fig. 3). CEUS showed no enhancement in five cases (Fig. 3) and epidermoid cysts were diagnosed.
3. Malignant germ cell tumor group: CDFI: Except for one case of embryonal carcinoma with poor blood flow signals (Fig. 4), the others all showed rich blood flow signals (Figs. 5, 6). Among them, five cases showed perforating vessels which tended to be diagnosed as lymphoma, and the rest were considered to be malignant, although the specific pathological type could not be determined. CEUS showed that 10 cases had heterogeneous high enhancement, with necrotic areas inside (Figs. 4, 5). With regards to fast forward and fast backward, three cases showed uniform high enhancement (two cases with fast forward and fast backward, one case with fast forward and slow backward). Heterogeneous hyperenhancement can be considered characteristic of malignant germ cell tumors. Two cases of spermatogenic tumors showed sparse and low enhancement and one case showed a large area without enhancement (Fig. 6). Among all the enhanced tumors, spermatogenic tumors were the only tumors with sparse and low enhancement, which could be regarded as its characteristic imaging appearance.
4. SLCT group: CDFI: five cases showed abundant blood flow signals, of which three cases showed peripheral annular blood flow (Fig. 7). In this study, peripheral annular blood flow was only seen in SLCT; this can therefore be regarded as its characteristic imaging appearance, leading to a diagnosis of SLCT. CEUS had characteristic features: all showed uniform high enhancement (no necrotic area) (Fig. 7), fast forward and slow backward.
5. Lymphoma group: CDFI: eight cases showed abundant blood flow signals (Fig. 8), three of which were diagnosed as lymphoma due to perforating vessels, and the rest were similar to malignant germ cell tumors, which were difficult to identify. CEUS: Except for one case with high enhancement and significant necrosis (perforating vessels in CDFI), the rest showed uniform high levels of enhancement (Fig. 8), fast forward and fast backward. Lymphoma was considered if the mass was larger and no necrotic areas were evident, comprehensive fast forward and fast backward, the patient was of older age and there were other manifestations (negative for tumor markers).

**Table 1:** Characteristics of conventional ultrasound in benign and malignant lesions of testis

Conventional ultrasound	Benign testicular lesions group	Malignant testicular tumors group	*p*-value
Age (years), mean ± SD	38.8 ± 14.9	47.4 ± 14.8	0.101
Lesion size (cm), mean ± SD	1.9 ± 0.9	4.6 ± 1.9	0.000
Boundary, n			
Clear	11	21	0.609
Unclear	2	2	
Echoes, n			
Hypoechoic	10	20	0.146
Isoechoic	3	1	
Cystic and solid mixed echoes	0	2	
Shape, n			
Round or oval	11	18	1.000
Irregular	2	5	
Echo distribution, n			
Homogenous	4	2	0.161
Heterogenous	9	21	
Blood flow, n			
None	4	0	0.001
Perforating vessels	0	8	
Peripheral annular	3	0	
Irregular	6	15	

*SD* standard deviation

**Table 2:** Characteristics of CEUS in benign and malignant lesions of testis

CEUS	Benign testicular lesions group	Malignant testicular tumors group	*p*-value
Enhancement, n			0.000
No enhancement	8	0	
Enhancement	5	23	

*CEUS* contrast-enhanced ultrasonography

The sensitivity, specificity, positive predictive value, negative predictive value and accuracy rates of conventional ultrasound for diagnosing benign testicular tumors based on "onion skin-like" echo (epidermoid cysts) and peripheral annular blood flow were 30.8%, 100.0%, 100.0%, 71.9% and 75.0%, respectively. According to CEUS without enhancement (non-neoplastic lesions and epidermoid cysts) and uniform high enhancement with fast forward and slow backward (SLCT), the sensitivity, specificity, positive predictive value, negative predictive value and accuracy rates for diagnosing benign testicular tumors were 100.0%, 100.0%, 100.0%, 100.0% and 100.0%, respectively. Compared with conventional ultrasound, these differences were statistically significant (*p* = 0.004).

## Discussion

Given that most testicular tumors are malignant germ cell tumors [3, 4], radical orchiectomy instead of biopsy is performed directly as biopsy could lead to a higher recurrence rate. However, generally performing radical orchiectomy will inevitably lead to overtreat (i.e., testis resection) some benign tumors and lymphomas. Non-neoplastic lesions of the testis normally do not require surgery, and most benign testicular tumors only require tumor enucleation. Lymphoma requires a comprehensive treatment that mainly involving chemotherapy and radiotherapy but not a surgical resection. Therefore, differentiating pathological types of testicular tumors by preoperative ultrasound (and not just a diagnosis as being benign or malignant) is of significant value in guiding surgical plan and preserving the testis of some patients. It is reported that the accuracy of CEUS in diagnosing benign and malignant testicular tumors is 88.9%, and the sample size is very small [8]. Literature reports showed that except for seminoma, other types of testicular tumors were very rare [8]. In our study, in addition to germ cell tumors, several other testicular lesions (such as non-neoplastic lesions, lymphoma, epidermoid cyst, SLCT, etc.) were also included. Our results showed that the accuracy of CEUS in diagnosing benign and malignant testicular tumors was 100.0%. CEUS not only accurately identified benign and malignant testicular lesions, but also well differentiated pathological types of testicular tumors, including testicular focal infarction, epidermoid cysts, spermatogenic tumors, SLTC and lymphoma.

### Testicular non-neoplastic lesions

Testicular non-neoplastic lesions include some focal infarcts and hematomas. The diagnosis of testicular hematoma is easier in the presence of a history of testicular trauma. However, a spontaneous testicular hematoma without a history of trauma is prone to be misdiagnosed as a testicular tumor [9]. In our study, one case of spontaneous hematoma had dull pain in the testis after bathing. Conventional ultrasound identified irregular hypoechoic signals in the right testis. Because there was no history of trauma, this was considered to be a malignant tumor. While CEUS showed no enhancement in the mass and a diagnosis of spontaneous hematoma was made. Ultrasound-guided aspiration was used to draw out blood and the mass was gradually absorbed and reduced during ultrasound follow-up; this was consistent with changes associated with a hematoma. Testicular necrosis resulting from complete testicular torsion is relatively easier to be diagnosed [10]. Focal testicular necrosis secondary to a history of orchitis or vascular injury can also be diagnosed by ultrasonography [11, 12]. Spontaneous testicular segmental infarcts are easily got misdiagnosed as tumors in the absence of a relevant medical history, leading to unnecessary surgical resection [13–15]. In our study, two cases of segmental infarction showed solid echoes on conventional ultrasound. Because there was no relevant medical history, conventional ultrasound could not determine whether these were tumors or not. While CEUS showed no enhancement, consistent with typical infarction. Due to the large infarct size, the infarct was resected. It is important to mention that there has been controversy on how to manage patients with segmental infarction due to a lack of clarity of the diagnosis and to prevent complications, but CEUS can help us diagnose it [15].

### Testicular epidermoid cysts

Testicular epidermoid cysts are a rare form of benign tumor that account for only 2.1% of all testicular tumors [16]. In recent years, testicular epidermoid cysts have been classified as prepubertal teratomas [17]. Usually these are asymptomatic or represent a palpable painless testicular mass. Unlike testicular malignancies, epidermoid cysts can be cured with only local excision [18]. However, it is still difficult to differentiate epidermoid cysts from malignant testicular tumors on imaging [17]. The conventional ultrasound appearance of a typical epidermoid cyst is an "onion skin-like" echo with a well-defined circular non-homogeneous echo with no blood flow signals on CDFI [19]. In this study, only one case was diagnosed with an epidermoid cyst due to an "onion skin-like" echo (no blood flow signal was seen). The remaining four cases were diagnosed as malignant tumors by conventional ultrasonography because of internal blood flow signals (possibly misdiagnosing the ‘twinkling artifact’ as blood flow). The “twinkling artifact” tends to occur behind irregular crystals (epidermoid cysts contain keratin and cholesterol crystals). If CEUS shows no enhancement, then the diagnosis is an epidermoid cyst. CEUS can help inexperienced sonographers identify twinkling artifacts to accurately diagnose epidermoid cysts and perform testicular-sparing local excision of the mass.

### Malignant germ cell tumors

Malignant germ cell tumors mainly include seminomas, embryonal carcinomas, spermatocytic tumors and mixed germ cell tumors [1]. Such tumors account for most testicular tumors and require radical orchiectomy [1, 4]. In our study, we found that malignant germ cell tumors were larger in size, which was due to their rapid growth rate. We speculate that small malignant germ cell tumors are difficult to be palpated by patients or physicians. In our study, the symptom of testicular tumors, whether benign or malignant, is mainly painless swelling of the scrotum. It is impossible to distinguish benign from malignant testicular tumors by clinical symptoms. Studies have shown that AFP and HCG have certain values in the diagnosis of testicular tumor types [1, 3]. Seminomas and spermatocytic tumors generally express normal tumor markers and a small number of seminomas have slightly elevated levels of HCG. AFP is known to be elevated in embryonal carcinomas and yolk sac tumors. The results of this study show that the increase of AFP and HCG values have certain value in the diagnosis of embryonal carcinoma and mixed germ cell tumors. The AFP and HCG values of most seminomas and spermatocytic tumors are normal, which is consistent with the literature reports [1, 3]. Malignant germ cell tumors show malignant features on conventional ultrasound (mostly with well-circumscribed hypoechoic and rich blood flow signals) but have similarities with lymphomas and SLCT. All cases in this group were diagnosed as malignant by conventional ultrasound, although the specific type was difficult to identify. Of these, five cases of malignant germ cell tumors showed perforating vessels. These cases tended to be diagnosed as lymphoma. However, CEUS showed heterogeneous hyperenhancement because of the obvious necrosis of embryonal carcinomas and mixed germ cell tumors, which is different from lymphoma and SLCT. Seven cases of seminoma showed heterogeneous enhancement, and three cases of uniform hyperenhancement were easily misdiagnosed as lymphoma. The age of the patients with such homogeneously enhanced seminoma did not exceed 48 years, and the median age of patients with primary testicular lymphomas was approximately 70 years [20]. The minimum age of the lymphoma patients in this group was 55 year. Thus, age can be used as a distinguishing feature. Spermatocytic tumors are a rare form of malignancy and account for 0.61% of all testicular germ cell tumors [21]. Spermatocytic tumors and seminomas have similar cytological appearances; thus, it is difficult to differentiate between seminoma and lymphoma with conventional ultrasound [22]. However, the performance of CEUS is different. Spermatocytic tumors are the only tumors with sparse and low enhancement (with obvious necrotic areas within) among all forms of enhanced tumors and can be regarded as the characteristic manifestation of spermatocytic tumors. This manifestation of spermatocytic tumors is related to myxoid degeneration, edema, hemorrhage and necrosis [23].

### SLCT

Most SLCTs are benign tumors [24]. To preserve the endocrine function of the testis, the general choice of treatment is enucleation of the testicular mass [25, 26]. Tumors are generally small, asymptomatic and are mostly detected by ultrasonography. A small number of patients present with gynecomastia and infertility, although when tumors are larger, they present with painless enlargement of the scrotum [27, 28]. In this study, no endocrine symptoms were detected in five cases (testosterone and estradiol assays were not performed), three cases were referred for a painless testicular mass, and two cases were found incidentally by ultrasound. Ultrasound findings were well-defined, round-like, isoechoic or hypoechoic, with abundant blood flow, similar to those of lymphoma and seminoma. In our study, peripheral annular blood flow was only seen in SLCT; therefore, this can be regarded as a characteristic manifestation. Accordingly, three cases were diagnosed with SLCT. CEUS has characteristic manifestations—all patients showed uniform high enhancement without necrosis, fast forward and slow backward. Comprehensive judgment can be made in combination with testosterone and estradiol measurements [28, 29].

### Testicular lymphoma

Primary testicular lymphomas are a rare form of extranodal lymphoma that accounts for 1–9% of all testicular malignancies [30, 31]. The age of onset is generally over 50 years and the median age at diagnosis of primary testicular lymphoma was reported to be 70 years [20]. The clinical manifestation was painless enlargement of the scrotum, which was a consistent observation throughout this study. In our study, the conventional ultrasonography findings of testicular lymphoma were round-like hypoechoic areas with clear boundaries and rich blood flow, similar to those of seminoma. It has been reported in the literature that a sign of perforating vessels (testicular long blood vessels run normally in the mass) has certain value in the diagnosis of testicular lymphoma [32]. However, in this study, only 37.5% (3/8) of lymphomas had perforating vessels, and five cases of malignant germ cell tumors had perforating vessels; thus, the diagnostic value of these perforating vessels is limited. With regards to CEUS, except for one case with high enhancement and significant necrosis (perforating vessels in CDFI), the other cases showed uniform high enhancement with fast forward and fast backward but without necrotic areas. Testicular lymphoma was considered if the mass was larger and no necrotic area was evident, fast forward and fast rewind, older age and negative tumor markers.

The present study had some limitations that needed to be considered. Firstly, this was a single-center retrospective study with a small sample size; thus, further research with a multi-center design and a large sample size is required. Secondly, since this study was limited by its retrospective design and differences in matched models, we did not perform CEUS-based quantitative analysis. In addition, the testicular lesions selected in this study were accompanied by pathological results. Therefore, many suspected non-neoplastic testicular lesions that did not undergo surgery were not included, resulting in certain selection bias. Moreover, because of the extremely high accuracy of the diagnosis of testicular tumors by the professional andrology sonographers in our center, they have won the trust of andrologists. Therefore, most testicular tumors have not undergone magnetic resonance examination; consequently, there is no other images available for comparative analysis.

In conclusion, CEUS could accurately distinguish between benign and malignant testicular tumors and could differentiate specific pathological types (such as testicular focal infarction, epidermoid cysts, spermatocytic tumors, SLTC and lymphomas). As different pathological types of testicular tumors had different treatment options, accurate preoperative diagnosis is critical for guiding the selection of appropriate treatment plans.

## Data Availability

All data generated or analysed during this study are included in this published article.
